# *rac*-Ethyl *rel*-(2*R*,3*R*,4*S*)-4-hy­droxy-1,2-dimethyl-5-oxopyrrolidine-3-carboxyl­ate

**DOI:** 10.1107/S2414314623000755

**Published:** 2023-02-09

**Authors:** Fatin Nur Ain Abdul Rashid, Muhamad Zulfaqar Bacho, Muhamad Azwan Hamali, Alexandra M. Z. Slawin, Mohd Fazli Mohammat, Ruwaida Shamsujunaidi, Mohd Abdul Fatah Abdul Manan

**Affiliations:** a Organic Synthesis Research Laboratory, Institute of Science, Universiti Teknologi MARA, 42300 Bandar Puncak Alam, Selangor, Malaysia; b Faculty of Applied Sciences, Universiti Teknologi MARA, 40450 Shah Alam, Selangor, Malaysia; c EaStCHEM School of Chemistry, University of St Andrews, North Haugh, St Andrews, KY16 9ST, United Kingdom; Benemérita Universidad Autónoma de Puebla, México

**Keywords:** crystal structure, oxopyrrolidine, ring conformation, hydrogen bonds

## Abstract

The asymmetric unit of the title compound, C_9_H_15_NO_4_, consists of a functionalized pyrrolidine ring having an envelope conformation, synthesized as an ethyl ester. The mol­ecule has three chiral centres and crystallized as a racemic mixture. In the crystal, mol­ecules are linked by pairwise O—H⋯O bonds, generating dimers with twofold rotational symmetry.

## Abstract 2

The crystal structure of the title compound exhibits an envelope conformation for the pyrrolidine ring, which includes three chiral centres.

## Graphical abstract

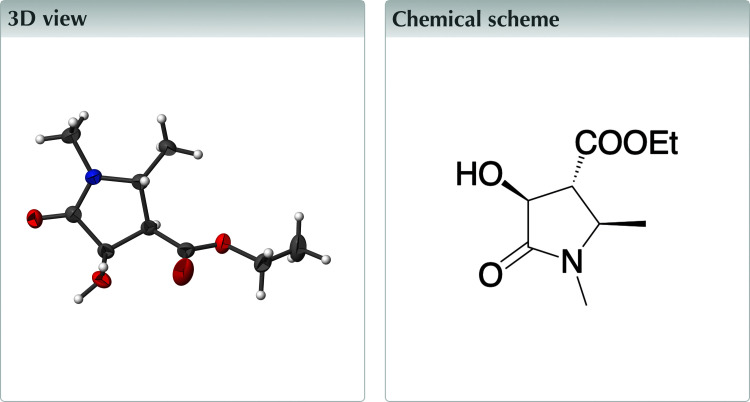

## Structure description

The heterocyclic compound 2-oxopyrrolidine and its derivatives have generated a lot of inter­est because of their practical significance (Pandya & Desai, 2020 ▸). These compounds have shown to be effective analgesics, anti-inflammatory (Salgın-Gökşen *et al.*, 2007 ▸), anti­viral (Tian *et al.*, 2009 ▸), anti­microbial (Özkay *et al.*, 2010 ▸; Salgın-Gökşen *et al.*, 2007 ▸), anti­tumor (Abdel-Aziz *et al.*, 2021 ▸), anti­convulsant (Angelova *et al.*, 2016 ▸), anti­depressant (Kulandasamy *et al.*, 2009 ▸), cardioprotective (Ghazouani *et al.*, 2019 ▸) and anti­platelet agents (Mashayekhi *et al.*, 2013 ▸; Ghazouani *et al.*, 2019 ▸).

During the course of our study towards pyrrolidine-based imino­sugars, we have synthesized the title compound by reduction of 2,3-dioxopyrrolidine (Bacho *et al.*, 2020 ▸; Abdul Rashid *et al.*, 2020 ▸). The starting material, 2,3-dioxopyrrolidine, was initially prepared *via* a multicomponent reaction, according to a previously reported procedure (Mohammat *et al.*, 2009 ▸, 2011 ▸).

**Figure 1:**
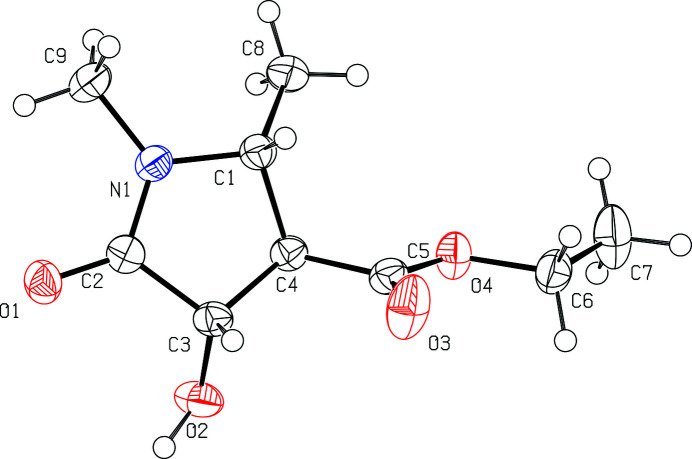
Crystal structure of the title compound, showing the atom-labelling scheme and displacement ellipsoids at the 50% probability level.

The title compound crystallizes in the monoclinic crystal system, space group *C*2/*c*, with one mol­ecule in the asymmetric unit (Fig. 1 ▸). The pyrrolidine ring (C1–C4/N1) adopts an envelope conformation, with atom C4 deviating by 0.180 (1) Å from the mean plane. There are three chiral centres within the ring, at C4, with a C1—C4—C5—O4 torsion angle of −94.04 (11)°. The methyl and hydroxyl groups, attached to C1 and C3, respectively, are orientated awayfrom the mean plane with C2—N1—C1—C8 and N1—C2—C3—O2 torsion angles of 142.07 (10) and −135.48 (10)°, respectively. Meanwhile, the ethyl ester group (O3/C5/O4/C6/C7) occupies the equatorial position on the pyrrolidine ring at C1, C3, and C4, . All bond lengths (Allen *et al.*, 1987 ▸) and angles in the mol­ecule show normal values.

**Figure 2:**
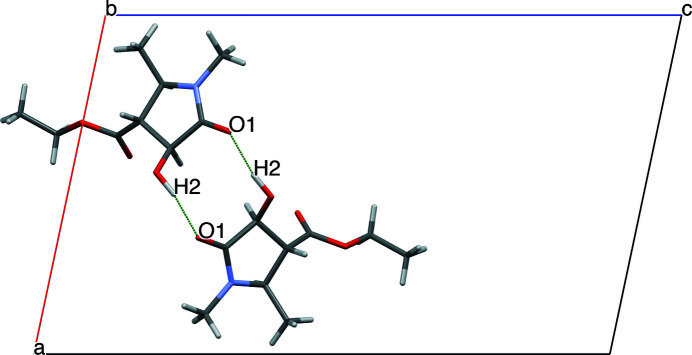
The O—H⋯O hydrogen bonds, indicated by green dashed bonds, forming 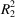 (10) motifs in the crystal.

**Figure 3:**
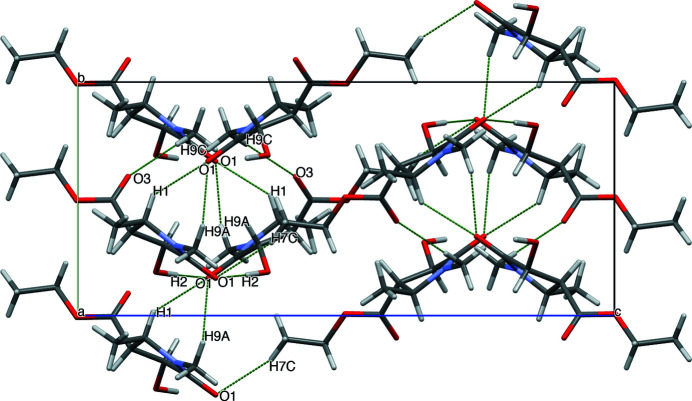
The mol­ecular packing of title compound, viewed down the *a* axis. Inter­molecular hydrogen bonds are indicated by green dashed lines.

In the crystal, the mol­ecules are linked by pairwise O—H⋯O hydrogen bonds, involving the carbonyl and hy­droxy groups, forming centrosymmetric 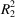 (10) ring motifs (Table 1 ▸, entry 1; Fig. 2 ▸). The packing also features C—H⋯O hydrogen bonds (Table 1 ▸), forming zigzag motifs propagating along the *c-*axis direction (Fig. 3 ▸).

## Synthesis and crystallization

A solution of 2,3-dioxopyrrolidine (2.00 g, 10.04 mmol) together with Pd—C (10% wt; 1.39 g, 1.31 mmol) and acetic acid (4.59 ml, 80.32 mmol) was stirred in ethanol. The reaction was stirred vigorously under a hydrogen atmosphere to completion (24 h) and then filtered through Celite. After removal of the solvent, the crude product was purified by flash column chromatography on silica gel using ethyl acetate/petroleum ether (9/1), to afford two compounds; *trans-*hy­droxy­ester **1** as a white solid and *cis-*hy­droxy­ester **2** as a colourless oil. The white solid of *trans*-hydroxyester **1** was recrystallized from methanol solution to give single crystals of the title compound **1** (0.24 g, 12%).

*trans-*hy­droxy­ester **1**: ^1^H NMR (400 MHz, CDCl_3_): δ 4.57 (*d*, *J* = 8.5 Hz, 1H), 4.22 (*q*, *J* = 6.9 Hz, 2H), 3.63 (*s*, 1H), 2.82 (*s*, 3H), 2.67 (*t*, *J* = 8.4 Hz, 1H), 1.37 (*d*, *J* = 3.7 Hz, 3H), 1.29 (*t*, *J* = 6.9 Hz, 3H); ^13^C NMR (100 MHz, CDCl_3_): δ 173.00 (C=O), 171.54 (C=O), 72.26 (CHOH), 61.71 (OCH_2_), 54.35 (CH), 31.23 (CHCH_3_), 27.33 (CH_3_N), 19.31 (CH_3_), 14.27 (CH_3_); GCMS *m*/*z* (EI, +ve): found: 201.10 ([*M*]^+^), calculated for C_9_H_15_NO_4_: 201.10.

*cis-*hy­droxy­ester **2**: (0.50 g, 25%). ^1^H NMR (400 MHz, CDCl_3_): δ 4.44 (*d*, *J* = 7.3 Hz, 1H), 4.19 (*td*, *J* = 7.2, 4.9 Hz, 2H), 3.74 (*t*, *J* = 6.6 Hz, 1H), 3.38 (*t*, *J* = 6.6 Hz, 1H), 2.82 (*s*, 3H), 1.32–1.23 (*m*, 6H); ^13^C NMR (100 MHz, CDCl_3_): δ 172.82 (C=O), 169.59 (C=O), 70.88 (CHOH), 61.11 (OCH_2_), 53.06 (CH), 49.21 (CHCH_3_), 27.13 (CH_3_N), 15.28 (CH_3_), 14.37 (CH_3_); GCMS *m*/*z* (EI, +ve): found: 201.10 ([*M*]^+^), calculated for C_9_H_15_NO_4_: 201.10.

## Refinement

Crystal data, data collection and structure refinement details are summarized in Table 2 ▸.

## Supplementary Material

Crystal structure: contains datablock(s) I. DOI: 10.1107/S2414314623000755/bh4072sup1.cif

Structure factors: contains datablock(s) I. DOI: 10.1107/S2414314623000755/bh4072Isup2.hkl

Supporting information file. DOI: 10.1107/S2414314623000755/bh4072Isup3.mol

Supporting information file. DOI: 10.1107/S2414314623000755/bh4072Isup4.cml

CCDC reference: 2238498

Additional supporting information:  crystallographic information; 3D view; checkCIF report

## Figures and Tables

**Table 1:** Hydrogen-bond geometry (Å, °)

*D*—H⋯*A*	*D*—H	H⋯*A*	*D*⋯*A*	*D*—H⋯*A*
O2—H2⋯O1^i^	0.97 (1)	1.78 (1)	2.7405 (12)	170 (2)
C1—H1⋯O1^ii^	1.00	2.62	3.3953 (14)	134
C9—H9*A*⋯O1^ii^	0.98	2.51	3.3355 (16)	142
C9—H9*C*⋯O3^iii^	0.98	2.54	3.5086 (15)	169
C7—H7*C*⋯O1^iv^	0.98	2.58	3.5134 (17)	160

Symmetry codes: (i) 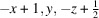 ; (ii) 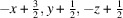 ; (iii) 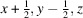 ; (iv) 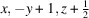 .

**Table 2:** Experimental details

Crystal data	
Chemical formula	C_9_H_15_NO_4_
*M*_r_	201.22
Crystal system, space group	Monoclinic, *C*2/*c*
Temperature (K)	173
*a*, *b*, *c* (Å)	12.1599 (15), 8.6065 (8), 20.217 (2)
β (°)	101.960 (3)
*V* (Å^3^)	2069.9 (4)
*Z*	8
Radiation type	Mo *K*α
μ (mm^−1^)	0.10
Crystal size (mm)	0.2 × 0.2 × 0.1

Data collection	
Diffractometer	Rigaku XtaLAB P200
Absorption correction	Multi-scan (*REQAB*; Rigaku, 1998 ▸)
*T*_min_, *T*_max_	0.879, 0.990
No. of measured, independent and observed [*I* > 2σ(*I*)] reflections	11140, 1874, 1769
*R*_int_	0.019
(sin θ/λ)_max_ (Å^−1^)	0.603

Refinement	
*R*[*F* ^2^ > 2σ(*F* ^2^)], *wR*(*F* ^2^), *S*	0.033, 0.088, 1.04
No. of reflections	1874
No. of parameters	134
No. of restraints	1
H-atom treatment	H atoms treated by a mixture of independent and constrained refinement
Δρ_max_, Δρ_min_ (e Å^−3^)	0.24, −0.18

Computer programs: *CrystalClear-SM Expert* (Rigaku, 2015 ▸), *SHELXT2018/2* (Sheldrick, 2015*a* ▸), *SHELXL2018/3* (Sheldrick, 2015*b* ▸) and *OLEX2* (Dolomanov *et al.*, 2009 ▸).

## full crystallographic data

### Crystal data

**Table:**

C_9_H_15_NO_4_	*F*(000) = 864
*M**_r_* = 201.22	*D*_x_ = 1.291 Mg m^−^^3^
Monoclinic, *C*2/*c*	Mo *K*α radiation, λ = 0.71075 Å
*a* = 12.1599 (15) Å	Cell parameters from 3484 reflections
*b* = 8.6065 (8) Å	θ = 2.1–27.5°
*c* = 20.217 (2) Å	µ = 0.10 mm^−^^1^
β = 101.960 (3)°	*T* = 173 K
*V* = 2069.9 (4) Å^3^	Prism, colorless
*Z* = 8	0.2 × 0.2 × 0.1 mm

### Data collection

**Table:**

Rigaku XtaLAB P200 diffractometer	1769 reflections with *I* > 2σ(*I*)
Detector resolution: 5.814 pixels mm^-1^	*R*_int_ = 0.019
ω scans	θ_max_ = 25.4°, θ_min_ = 2.1°
Absorption correction: multi-scan (*REQAB*; Rigaku, 1998)	*h* = −14→14
*T*_min_ = 0.879, *T*_max_ = 0.990	*k* = −10→10
11140 measured reflections	*l* = −24→24
1874 independent reflections	

### Refinement

**Table:**

Refinement on *F*^2^	Primary atom site location: dual
Least-squares matrix: full	Secondary atom site location: difference Fourier map
*R*[*F*^2^ > 2σ(*F*^2^)] = 0.033	Hydrogen site location: mixed
*wR*(*F*^2^) = 0.088	H atoms treated by a mixture of independent and constrained refinement
*S* = 1.04	*w* = 1/[σ^2^(*F*_o_^2^) + (0.0452*P*)^2^ + 1.1669*P*] where *P* = (*F*_o_^2^ + 2*F*_c_^2^)/3
1874 reflections	(Δ/σ)_max_ < 0.001
134 parameters	Δρ_max_ = 0.24 e Å^−^^3^
1 restraint	Δρ_min_ = −0.18 e Å^−^^3^

### Special details

**Table:**

Refinement. The hydroxyl H atom (H2) was refined with free coordinates and isotropic displacement parameter.

### Fractional atomic coordinates and isotropic or equivalent isotropic displacement parameters (Å^2^)

**Table:**

	*x*	*y*	*z*	*U*_iso_*/*U*_eq_	
O1	0.65691 (7)	0.16087 (10)	0.24315 (4)	0.0360 (2)	
O2	0.53577 (7)	0.18679 (11)	0.35435 (4)	0.0388 (2)	
O3	0.58426 (9)	0.59491 (13)	0.40623 (5)	0.0556 (3)	
O4	0.68278 (7)	0.49308 (10)	0.50163 (4)	0.0348 (2)	
N1	0.78865 (8)	0.29976 (11)	0.31723 (5)	0.0282 (2)	
C2	0.68560 (9)	0.24451 (13)	0.29333 (5)	0.0275 (3)	
C3	0.60731 (9)	0.30292 (13)	0.33799 (5)	0.0278 (3)	
H3	0.561557	0.391982	0.315420	0.033*	
C4	0.68834 (9)	0.35965 (13)	0.40091 (5)	0.0259 (3)	
H4	0.706096	0.271938	0.433837	0.031*	
C1	0.79582 (9)	0.40440 (13)	0.37550 (5)	0.0275 (3)	
H1	0.789576	0.514521	0.359380	0.033*	
C9	0.88156 (10)	0.28015 (15)	0.28261 (6)	0.0359 (3)	
H9A	0.902826	0.381524	0.267070	0.043*	
H9B	0.858357	0.211119	0.243670	0.043*	
H9C	0.946000	0.234634	0.313807	0.043*	
C5	0.64421 (9)	0.49526 (13)	0.43501 (6)	0.0294 (3)	
C6	0.64892 (11)	0.62159 (15)	0.54030 (6)	0.0364 (3)	
H6A	0.566573	0.619870	0.537083	0.044*	
H6B	0.669524	0.722225	0.522480	0.044*	
C7	0.70872 (15)	0.60126 (19)	0.61170 (7)	0.0567 (4)	
H7A	0.790035	0.600863	0.614059	0.068*	
H7B	0.686220	0.502495	0.629065	0.068*	
H7C	0.689349	0.687011	0.639073	0.068*	
C8	0.90310 (10)	0.38489 (16)	0.42851 (6)	0.0374 (3)	
H8A	0.967321	0.419643	0.410005	0.045*	
H8B	0.912839	0.275194	0.441383	0.045*	
H8C	0.898462	0.447241	0.468402	0.045*	
H2	0.4729 (10)	0.174 (2)	0.3162 (6)	0.063 (5)*	

### Atomic displacement parameters (Å^2^)

**Table:**

	*U*^11^	*U*^22^	*U*^33^	*U*^12^	*U*^13^	*U*^23^
O1	0.0353 (5)	0.0407 (5)	0.0292 (4)	0.0041 (4)	0.0005 (3)	−0.0061 (4)
O2	0.0307 (4)	0.0512 (5)	0.0333 (5)	−0.0129 (4)	0.0038 (4)	0.0043 (4)
O3	0.0666 (7)	0.0579 (7)	0.0377 (5)	0.0363 (5)	0.0007 (5)	−0.0005 (4)
O4	0.0418 (5)	0.0345 (5)	0.0271 (4)	0.0084 (4)	0.0048 (3)	−0.0033 (3)
N1	0.0256 (5)	0.0321 (5)	0.0274 (5)	0.0024 (4)	0.0067 (4)	0.0004 (4)
C2	0.0280 (5)	0.0283 (6)	0.0247 (5)	0.0033 (4)	0.0022 (4)	0.0041 (4)
C3	0.0239 (5)	0.0324 (6)	0.0262 (6)	0.0001 (4)	0.0031 (4)	0.0030 (4)
C4	0.0238 (5)	0.0286 (6)	0.0247 (5)	0.0024 (4)	0.0034 (4)	0.0025 (4)
C1	0.0257 (5)	0.0273 (5)	0.0296 (6)	0.0003 (4)	0.0061 (4)	−0.0003 (4)
C9	0.0297 (6)	0.0449 (7)	0.0353 (6)	0.0061 (5)	0.0118 (5)	0.0014 (5)
C5	0.0248 (5)	0.0342 (6)	0.0291 (6)	0.0029 (5)	0.0054 (4)	0.0014 (5)
C6	0.0399 (7)	0.0341 (6)	0.0370 (7)	0.0036 (5)	0.0126 (5)	−0.0060 (5)
C7	0.0757 (10)	0.0528 (9)	0.0374 (7)	0.0168 (8)	0.0023 (7)	−0.0136 (6)
C8	0.0252 (6)	0.0478 (7)	0.0372 (6)	−0.0010 (5)	0.0021 (5)	−0.0065 (5)

### Geometric parameters (Å, º)

**Table:**

O1—C2	1.2340 (14)	C1—H1	1.0000
O2—C3	1.4087 (14)	C1—C8	1.5162 (16)
O2—H2	0.973 (5)	C9—H9A	0.9800
O3—C5	1.1957 (14)	C9—H9B	0.9800
O4—C5	1.3313 (14)	C9—H9C	0.9800
O4—C6	1.4620 (14)	C6—H6A	0.9900
N1—C2	1.3337 (15)	C6—H6B	0.9900
N1—C1	1.4709 (14)	C6—C7	1.4860 (19)
N1—C9	1.4569 (14)	C7—H7A	0.9800
C2—C3	1.5266 (15)	C7—H7B	0.9800
C3—H3	1.0000	C7—H7C	0.9800
C3—C4	1.5192 (15)	C8—H8A	0.9800
C4—H4	1.0000	C8—H8B	0.9800
C4—C1	1.5488 (14)	C8—H8C	0.9800
C4—C5	1.5095 (15)		
			
C3—O2—H2	108.5 (10)	N1—C9—H9B	109.5
C5—O4—C6	116.81 (9)	N1—C9—H9C	109.5
C2—N1—C1	113.81 (9)	H9A—C9—H9B	109.5
C2—N1—C9	123.27 (10)	H9A—C9—H9C	109.5
C9—N1—C1	122.17 (9)	H9B—C9—H9C	109.5
O1—C2—N1	126.17 (10)	O3—C5—O4	123.63 (11)
O1—C2—C3	125.01 (10)	O3—C5—C4	124.81 (10)
N1—C2—C3	108.82 (9)	O4—C5—C4	111.53 (9)
O2—C3—C2	113.35 (10)	O4—C6—H6A	110.3
O2—C3—H3	109.8	O4—C6—H6B	110.3
O2—C3—C4	110.88 (9)	O4—C6—C7	107.16 (10)
C2—C3—H3	109.8	H6A—C6—H6B	108.5
C4—C3—C2	103.02 (8)	C7—C6—H6A	110.3
C4—C3—H3	109.8	C7—C6—H6B	110.3
C3—C4—H4	109.1	C6—C7—H7A	109.5
C3—C4—C1	104.33 (8)	C6—C7—H7B	109.5
C1—C4—H4	109.1	C6—C7—H7C	109.5
C5—C4—C3	113.62 (9)	H7A—C7—H7B	109.5
C5—C4—H4	109.1	H7A—C7—H7C	109.5
C5—C4—C1	111.32 (9)	H7B—C7—H7C	109.5
N1—C1—C4	101.50 (8)	C1—C8—H8A	109.5
N1—C1—H1	109.4	C1—C8—H8B	109.5
N1—C1—C8	113.44 (9)	C1—C8—H8C	109.5
C4—C1—H1	109.4	H8A—C8—H8B	109.5
C8—C1—C4	113.55 (9)	H8A—C8—H8C	109.5
C8—C1—H1	109.4	H8B—C8—H8C	109.5
N1—C9—H9A	109.5		
			
O1—C2—C3—O2	45.04 (15)	C1—N1—C2—O1	176.42 (11)
O1—C2—C3—C4	164.92 (11)	C1—N1—C2—C3	−3.06 (12)
O2—C3—C4—C1	148.36 (9)	C1—C4—C5—O3	84.29 (14)
O2—C3—C4—C5	−90.22 (11)	C1—C4—C5—O4	−94.04 (11)
N1—C2—C3—O2	−135.48 (10)	C9—N1—C2—O1	6.15 (18)
N1—C2—C3—C4	−15.60 (12)	C9—N1—C2—C3	−173.33 (10)
C2—N1—C1—C4	19.89 (12)	C9—N1—C1—C4	−169.72 (9)
C2—N1—C1—C8	142.07 (10)	C9—N1—C1—C8	−47.54 (14)
C2—C3—C4—C1	26.79 (11)	C5—O4—C6—C7	−175.57 (11)
C2—C3—C4—C5	148.21 (9)	C5—C4—C1—N1	−151.06 (9)
C3—C4—C1—N1	−28.13 (10)	C5—C4—C1—C8	86.83 (11)
C3—C4—C1—C8	−150.24 (10)	C6—O4—C5—O3	−0.29 (17)
C3—C4—C5—O3	−33.14 (16)	C6—O4—C5—C4	178.06 (9)
C3—C4—C5—O4	148.53 (9)		

### Hydrogen-bond geometry (Å, º)

**Table:**

*D*—H···*A*	*D*—H	H···*A*	*D*···*A*	*D*—H···*A*
O2—H2···O1^i^	0.97 (1)	1.78 (1)	2.7405 (12)	170 (2)
C1—H1···O1^ii^	1.00	2.62	3.3953 (14)	134
C9—H9*A*···O1^ii^	0.98	2.51	3.3355 (16)	142
C9—H9*C*···O3^iii^	0.98	2.54	3.5086 (15)	169
C7—H7*C*···O1^iv^	0.98	2.58	3.5134 (17)	160

Symmetry codes: (i) −*x*+1, *y*, −*z*+1/2; (ii) −*x*+3/2, *y*+1/2, −*z*+1/2; (iii) *x*+1/2, *y*−1/2, *z*; (iv) *x*, −*y*+1, *z*+1/2.
